# Enhancing the Glucose Flux of an Engineered EP-Bifido Pathway for High Poly(Hydroxybutyrate) Yield Production

**DOI:** 10.3389/fbioe.2020.517336

**Published:** 2020-08-27

**Authors:** Ying Li, Zhijie Sun, Ya Xu, Yaqi Luan, Jiasheng Xu, Quanfeng Liang, Qingsheng Qi, Qian Wang

**Affiliations:** ^1^National Glycoengineering Research Center, State Key Laboratory of Microbial Technology, Shandong University, Qingdao, China; ^2^Marine Biology Institute, Shantou University, Shantou, China; ^3^CAS Key Lab of Biobased Materials, Qingdao Institute of Bioenergy and Bioprocess Technology, Chinese Academy of Sciences, Qingdao, China

**Keywords:** poly(hydroxybutyrate) yield, glucose flux, EP-bifido pathway, MAGE, *Escherichia coli*

## Abstract

**Background:**

As the greenhouse effect becomes more serious and carbon dioxide emissions continue rise, the application prospects of carbon sequestration or carbon-saving pathways increase. Previously, we constructed an EP-bifido pathway in *Escherichia coli* by combining Embden-Meyerhof-Parnas pathway, pentose phosphate pathway and “bifid shunt” for high acetyl-CoA production. There is much room for improvement in the EP-bifido pathway, including in production of target compounds such as poly(hydroxybutyrate) (PHB).

**Result:**

To optimize the EP-bifido pathway and obtain higher PHB yields, we knocked out the specific phosphoenolpyruvate phosphate transferase system (PTS) component II Cglc, encoded by *ptsG*. This severely inhibited the growth and sugar consumption of the bacterial cells. Subsequently, we used multiple automated genome engineering (MAGE) to optimize the ribosome binding site (RBS) sequences of *galP* (galactose: H (+) symporter) and *glk* (glucokinase gene bank: NC_017262.1), encoding galactose permease and glucokinase, respectively. Growth and glucose uptake were partially restored in the bacteria. Finally, we introduced the glf (UDP-galactopyranose) from *Zymomonas mobilis* mutase sugar transport vector into the host strain genome.

**Conclusion:**

After optimizing RBS of *galP*, the resulting strain L-6 obtained a PHB yield of 71.9% (mol/mol) and a 76 wt% PHB content using glucose as the carbon source. Then when *glf* was integrated into the genome strain L-6, the resulting strain M-6 reached a 5.81 g/L PHB titer and 85.1 wt% PHB content.

## Introduction

In 2018, global carbon dioxide emissions increased 1.7% over the previous year, hitting a record high of 33.143 billion tons. Accelerating the adoption of renewable energy and improving energy efficiency in response to global warming are urgent priorities (International Energy Agency, 2019). Bio-manufacturing, which uses food crops as raw materials, has wide application prospects. Through biological manufacturing, biomass resources can be converted to ethanol, polylactic acid, 1, 3-propanediol, and other bulk chemicals (Ragauskas et al., 2006; Lee et al., 2012). A key barrier to this process is the CO_2_ emissions that occur during natural aerobic fermentation. Many carbon dioxide fixation pathways have been exploited using the six carbon fixation pathways discovered in nature (Gong et al., 2016). However, complex reaction steps and enzyme requirements limit the broad application of these carbon sequestration pathways (Erb et al., 2007; Schwander et al., 2016).

**FIGURE 1:**
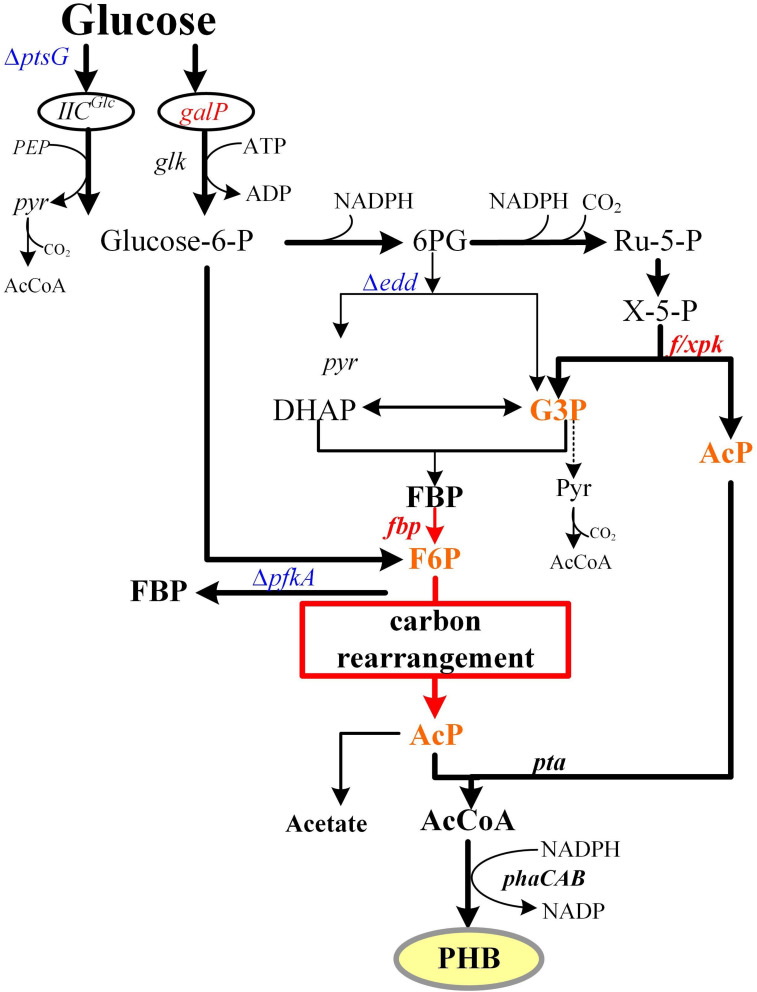
EP-bifido pathway and theory carbon flux distribution. Metabolic optimization of EP-bifido pathway. The red box referred to the carbon rearrangement procedure. Overexpressed genes are shown in red. Deleted genes are shown in blue. Intermediate metabolites participated in carbon rearrangement are shown in orange.

In natural microorganism fermentation, glucose can be transformed through the Embden-Meyerhof-Parnas (EMP) pathway to pyruvate. Pyruvate metabolism leads to the production of two molecules of acetyl-CoA (AcCoA), the key precursor of ethanol, butanol, fatty acids, amino acids, and pharmaceuticals. This process produces two molecules of CO_2_ from one mol of glucose, making it an uneconomical way to biosynthesize products using AcCoA as precursor. Other glycol metabolism pathways, such as the phosphoketolase pathway, employ a pentose phosphate specific transketolase to produce a mixture of ethanol, lactic acid, and CO_2_. Additionally, the bifido bacteria exclusive bifid shunt pathway can generate 1 mol of lactic acid and 1.5 mol of acetate from 1 mol of glucose (Meile et al., 2001; Posthuma et al., 2002). However, all of these glucose metabolic pathways lose carbon in the form of CO_2_ during the decarboxylation process. Therefore, several carbon-saving pathways, including the non-oxidative glycolytic (NOG) and EP-bifido pathways, have been engineered. The NOG pathway can transform all six carbon atoms of glucose into three AcCoA molecules without CO_2_ loss (Bogorad et al., 2013). However, it cannot provide the reducing power NADPH that is needed for PHB and other chemicals biosynthesis. The EP-bifido pathway employs EMP, pentose phosphate pathway (PPP) and the Bifido shunt for high-yield of AcCoA generation. As a reducing power sponsor, the oxidation part of the PPP consumes 1 mol of glucose, and provides 2 mol of NADPH and 1mol of xylulose-5-phosphate (X5P). The enzyme encoded by the *f/xpk* gene of the EP-bifido pathway has both fructose-6-phosphate (F6P) and X5P activity. It is able to catalyze X5P to form acetyl phosphate (AcP) and glyceraldehyde 3-phosphate (G3P) or split F6P to form erythrose 4-phosphate and AcP. The former G3P can generate AcP through carbon rearrangement, each of these processes releases only 1 mol of CO_2_, thus saving the carbon source to a certain extent (Figure 1). This carbon-saving pathway has been applied to the production of several compounds that use AcCoA as the precursor (Wang et al., 2019). Previously, we achieved a relatively high level of production and yield, but there remains room to improve carbon conversion in our system.

In *Escherichia coli*, glucose is transported through the phosphate transferase system (PTS). This system is involved in phosphoenolpyruvate (PEP)-dependent sugar transport and its activity has an important impact on carbon flux redistribution in the PEP and pyruvate nodes (Gosset, 2005). Glucose was phosphorylated to G6P by the phosphoryl generated from PEP, which was dephosphorylated to form pyruvate. Then pyruvate further decarboxylated to AcCoA and released 1 mol of CO_2_, leading to the loss of the carbon source. In addition, PEP is a key intermediate metabolite of the EMP pathway. Therefore, an increase in the EMP pathway reduces the carbon conversion efficiency of the EP-bifido pathway.

Poly(hydroxybutyrate) (PHB) is the most common poly(hydroxyalkanoate) (PHA). PHB can be synthesized and accumulated by more than 300 microorganisms as both an energy and carbon store (Lee and Choi, 2001; van der Walle et al., 2001). The *in vivo* biosynthesis of PHB requires three steps using AcCoA as the precursor, and PHB production has been intensively studied (Lee et al., 1994; Wang Q. et al., 2009). Many strategies have been applied to engineer *E. coli* to improve PHB production, however, the yield still has much room to progress. By overexpressing NAD kinase, recombinant *E. coli* produced 14 g/L PHB and the yield based on glucose reached 0.31 mol/mol (0.15 g PHB/g glucose) (Li et al., 2009). By applying fed-batch strategy, *E. coli* could accumulate 125 g/L PHB, but the yield based on glucose was only 0.46 mol/mol (0.22 g PHB/g glucose) (Mozumder et al., 2014). Previously, we have achieved relatively high level of PHB content and PHB yield (68.4 wt% and 63.7% mol/mol, respectively) (Wang et al., 2019). In this study, we optimized the EP-bifido pathway for improved PHB production in *E. coli*. The non-PTS glucose transport pathway genes *glk*, *galP* and heterogeneous *glf* were enhanced and introduced into EP-bifido strains through multiple automated genome engineering (MAGE) and conditional-replication, integration, excision, and retrieval (CRIM) plasmids. The improved PHB production indicates that our modification increased the efficiency of artificial carbon-saving pathways for high carbon conversion rate from glucose.

## Materials and Methods

### Culture Media and Conditions

For plasmid preparation, *E. coli* strains were cultured at 37°C on a rotary shaker (220 rpm) in test. For plasmid preparation, *E. coli* strains were cultured at 37°C on a rotary shaker (220 rpm) in test tubes containing 5 mL Lysogeny broth (LB) medium. For PHB biosynthesis, 50-mL shake flask cultures were started by 2% inoculation from the 5-mL LB culture. The 50-mL cultures contained M9 minimal medium with 0.2% yeast extract containing 20 g/L glucose and shaken at 37°C in a rotary shaker (120 rpm) for 48 h. Overnight cultures were shaken at 37°C in a rotary shaker (220 rpm). Antibiotics were added as follows: ampicillin (Amp) 100 μg/mL, spectinomycin (Spc) 50 μg/mL, and chloromycetin (Cm) 25 μg/mL. For MAGE procedure, strains were cultivated in SOB medium.

Lysogeny broth medium contains (g/L): tryptone (10), yeast extract (5), and NaCl (10). M9 medium contains (g/L): Na_2_HPO_4_⋅12H_2_O (15.138), KH_2_PO_4_ (3), NaCl (0.5), and NH_4_Cl (1). SOB medium contains (g/L): tryptone (20), yeast extract (5), and NaCl (5).

### Strains and Plasmids

All *E. coli* strains and plasmids used are listed in Table 1. DH-EP was used as the starting strain for further genetic manipulation. All primers used for molecular manipulations are listed in Table 2.

**TABLE 1:** Bacterial strains and plasmids used in this study.

Strain and plasmids	Relevant properties	Sources
**Strains**		
JW1087-2	JW25113 derivative, Δ*ptsG*:*kan*	Baba et al. (2006)
DH-EP	DH5α derivative, Δ*edd*Δ*pfkA*	Wang et al. (2019)
DH-EPP	DH5α derivative, Δ*edd*Δ*pfkA*Δ*ptsG*	This study
L-6	DH-EPP derivative, Δ*edd*Δ*pfkA*Δ*ptsG galP* RBS:TGAAAGGGAAA	This study
M-6	L-6 derivative, Δ*edd*Δ*pfkA*Δ*ptsG*:*trc-rbs-glf_*zm*_*	This study
**Plasmids**		
pCAB	pBluescriptII SK, phbC and phbAB gene from *Ralstonia eutropha*	Wang et al. (2019)
pCDFtrc	Cloning vector, Spe^R^	Wang et al. (2019)
pFF	pCDFtrc, *fxpk* gene from *B. adolescentis* and *fbp* gene from *E. coli*	Wang et al. (2019)
pKD3	Template plasmid with Cm^R^ gene and FLP recognition target	Datsenko and Wanner (2000)
pTKRED	P_BAD_ promoter containing plasmid, Spe^R^	Kuhlman and Cox (2010)
pCP20	Helper plasmid expressing FLP recombinase, ts-rep, Amp^R^, Cm^R^	Datsenko and Wanner (2000)
pAH69	Helper plasmid expressing HK022 integrase, Amp^R^	Haldimann and Wanner (2001)

**TABLE 2:** Key oligonucleotide primers used in this study for DNA manipulation.

Primers	Sequence (5′-3′)
Q-ptsG-F	5′-GGCTGTGTTGAAAGGTGTTGC-3′
Q-ptsG-R	5′-AACGCGCTATATTGCAGAGG-3′
Glf-F	5′-GGTCGGTAAATCGCTGCTTGACAATTAATCATCCGGC TCGTATAATGTCTAGAGAAAGAGGAGAAATACTAGATGAG TTCTGAAAGTAGTCAGGGTC-3′
Glf-R	5′-GCCTACCCGGATATTATCGTGAGGATGCGAATTGTG TAGGCTGGAGCTGCTTC-3′
R6K-F	5′-TCGCATCCTCACGATAATATCCGGGTAGGC-3′
R6K-R	5′-TTGTCAAGCAGCATCAGCGATTTACCGACCGATCC GGCCACGATGCGTCC-3′

The Red homologous recombination method was employed for gene deletion. The pTKRED complementary plasmid was transformed into the target strain. Deletion fragments of *ptsG* gene were amplified from the JW1087-2 single-gene knockout mutant (Baba et al., 2006) (bought from Coli Genetic Stock Center, CGSC) using primers Q-ptsG-F/Q-ptsG-R.

### Measurement of Extracellular Metabolites

A spectrophotometer was used to measure the optical density at 600 nm (OD_600_) of the bacterial culture. PHB was quantified using gas chromatography (GC). Cells were harvested by centrifugation at 6,000 × *g* for 10 min, 4°C. The cell pellets were washed twice with distilled water and lyophilized for 7 h. Before GC analysis, 1 mL chloroform, 850 μL methanol, and 150 μL sulfuric acid (98%, w/w) were added to the weighed cells in vials. The vials were incubated at 100°C for 1 h. Then, 1 mL water was added for stratification and cooling vials. After standing for 1 h, the mixture separated into layers and the heavier chloroform phase was transferred to new vial for GC analysis. The GC detection process was performed using a Shimadzu GC2010 gas chromatograph (Kyoto, Japan) equipped with an AOC-20i auto injector and a Restek Rtx-5 column. PHB standard samples of methyl-(R)-3-hydroxybutyrate (Sigma-Aldrich) were dissolved in chloroform and analyzed by GC. The temperature program used was: 80°C for 1 min, ramped to 120°C at 10°C/min, then ramped to 160°C at 45°C/min for 5 min, and the total time was 10.89 min.

For extracellular metabolite analysis, 1 mL of culture was centrifuged at12,000 × *g* for 2 min. The supernatant was filtered through a 0.22-μm syringe filter for high-performance liquid chromatography analysis. Glucose, acetate, and pyruvate were measured on an ion exchange column (HPX-87H; Bio-Rad Labs) with a differential refractive index detector (Shimadzu RID-10A). A 0.5-mL/min mobile phase using a 5-mM H_2_SO_4_ solution was applied to the column. The column was operated at 65°C.

### MAGE Procedure

The ribosome binding sites (RBSs) designed for the modulation of *GalP* and *glk* transcription rates were 5′-GTCGTACTC ACCTATCTTAATTCACAATAAAAAATAACCADDRRRRRD DDDATCATGCCTGACGCTAAAAAACAGGGGCGGTCAAA CAAG-3′ (D = A, G, T; and R = A, G) and 5′-GCCGCCCACA TCACCGACTAATGCATACTTTGTCATTCTHHHHYYYYYH HGCTAAAGTCAAAATAATTCTTTCTCACACTGTAAATAC CT-3′ (H = T, C, A; and Y = T, C), respectively, with four phosphorothioated bases at the 5′ terminus. The initiation of MAGE requires that pTKRED was transformed into the target strain. The MAGE cycles were performed by growing DH-EPP in 5 mL SOB medium at 30°C and shaking at 220 rpm for 12 h. For the first MAGE round, 5-mL shake flask cultures using SOB broth were started with a 1% inoculation from the overnight culture. Isopropyl-β-D-thiogalactopyranoside (IPTG) was added to a final concentration of 0.5 mM to induce λ-prophage (*bet*, *gam*, and *exo*) gene expression. Cells were then incubated at 30°C and shaking at 220 rpm until reaching an OD_600_ of 0.5 to 0.6. Cells were collected (2 mL), pelleted, and washed three times with cold sterile water to make them electrocompetent. ssDNA mixture (1 μM) was added to electrocompetent cells and electroporated at 2.5 kV. To start the second MAGE round, cells were recovered in 5 mL SOC with IPTG until their OD_600_ reached 0.5 to 0.6, after which cells underwent pelleting, washing, and electroporation. Three to four MAGE rounds were performed per day and 16 cycles were performed in total. The resulting pool of variants were then characterized using the Nile red assay.

### Screening of PHB Competent Cells by Nile Red Assay

When PHB is combined with Nile red dye a red color is produced. We transformed the pCAB plasmid into these variants and added 100 μL Amp, 50 μL IPTG, 20 g/L glucose, and 200 μL Nile red dye to the solid M9 medium supplemented with 0.2% yeast extract. The MAGE variants were diluted 200-fold and spread onto several plates. After incubation at 37°C for 16 h, the plates were placed at 4°C for 3 days to allow the color reaction to develop. Based on the color difference, we picked single red colonies for sequencing. For all the sequenced colonies with mutations identified, the pTKRED plasmid was removed and the strains were transformed with the pFF and pCAB plasmids for further verification of the PHB competent cells.

### Integration of glf

For glf integration, the *trc-rbs-glf* module was amplified from *Zymomonas mobilis* genomic DNA by PCR using primers GLF-F and GLF-R. The PCR product (*trc-rbs-glf*) was cloned into a vector that carries R6K replicon and phage attachment sites (attP). This plasmid was named R6K-glf and was confirmed by DNA sequencing. The pAH69 helper plasmid was transformed into the L-6 strain. The target strain carrying pAH69 was incubated overnight at 30°C and transferred to 37°C for 1 h before transfection. Then the pR6K-glf plasmid was introduced into L-6 by electroporation. The centrifuged bacteria were plated onto plates containing 25 μg/mL kanamycin for overnight incubation at 37°C. R6K-glf positive transformants were selected by their kan^R^ phenotype and were verified by PCR.

### Determination of CO_2_ Emissions

CO_2_ emission was determined using a thermostatic oscillation incubator with a CO_2_ detector (BCP-CO_2_, Bluesens, Germany) that monitored CO_2_ volume every 20 s and transmitted the data to a computer. Cultures were grown at 37°C with shaking at 150 rpm.

## Results

### Inhibiting PTS to Reduce PEP Consumption

**FIGURE 2:**
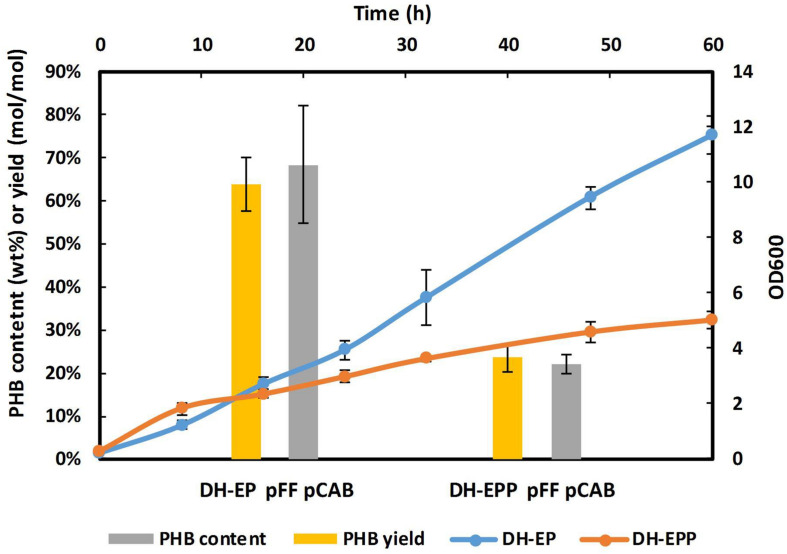
Fermentation result of *ptsG* knockout strain. The date of PHB yield and content of both strains were calculated based on fermentation samples at 60 h. The time axis at the top was used to depict growth curve. The growth carve was showed in orange red. The experiments were performed in duplicate and error bars indicate s.d. Strains were cultivated with M9 at 37°C, 150 rpm.

In *E. coli*, glucose is transported through the PTS system. This system is involved in PEP-dependent sugar transport and its activity has an important impact on carbon flux redistribution in the PEP and pyruvate nodes (Gosset, 2005). Glucose transport into the cytoplasm by EIICB^Glc^ (encoded by *ptsG*) is coupled to its phosphorylation. The phosphate group is derived from PEP and is transferred via a cascade of proteins, enzyme I (EI), HPr, EIIA, and EIIB. This procedure consumes almost half of the PEP (Valle et al., 1996; Wang et al., 2012). Glucose was phosphorylated to G6P by phosphoryl generated from PEP dephosphorylated to pyruvate, the formed pyruvate is further decarboxylated to AcCoA and releases 1 mol CO_2_, leading to carbon source loss. While PEP is not the precursor in our study, PEP consumption would convert carbon flux to the EMP pathway, which is not desirable in our EP-bifido pathway. Therefore, modulation of the PEP-independent uptake and phosphorylation system is required. Knocking out *ptsG* and replacing it with other glucose transport pathways is a common method used in the production of PEP-precursor products (Gosset, 2005; Lee et al., 2005; Li et al., 2013; Kyselova et al., 2018). Therefore, we knocked out *ptsG* in strain DH-EP and named the strain DH-EPP. But found that *ptsG* deletion severely impaired the growth capacity of the resulting strain. Compared with DH-EP (pFFpCAB) strain, PHB yield of DH-EPP strain decreased from 63.7 to 26.3% (mol/mol) (Figure 2).

For glucose uptake recovery, replacing PTS with an alternative PEP-independent uptake and phosphorylation system could be an efficient solution to this problem.

### Improving Glucose Flux Through Non-PTS Pathway

When *E. coli* strains lack PTS, the low affinity galactose: H^+^ symporter, GalP, encoded by *galP*, is induced. Glucose internalized by GalP must be phosphorylated by glucokinase (encoded by *glk*), which catalyzes the ATP-dependent phosphorylation of glucose to generate G6P in the cytoplasm without CO_2_ emission, thus saving the carbon source (Gosset, 2005). Therefore, we modified *galP* and *glk* expression levels to improve the glucose utilization rate. While increasing *galP* and *glk* transcriptional levels through plasmid overexpression is a pervasive strategy (Hernandez-Montalvo et al., 2003; Wang et al., 2006), it has several disadvantages, including metabolic burden and unexpected lateral effects. Modulation of *galP* and *glk* expression levels through plasmid overexpression or high strength promoter substitution cannot provide multiple combination of expression intensity for screening. Discovering a way to effectively modulate the transcription of the two genes to an optimal strength in combination is a pressing problem. We adopted MAGE to simultaneously regulate *galP* and *glk* expression levels (Wang H.H. et al., 2009). Using this approach, colonies with high PHB yields can be identified using Nile red dye staining.

**FIGURE 3:**
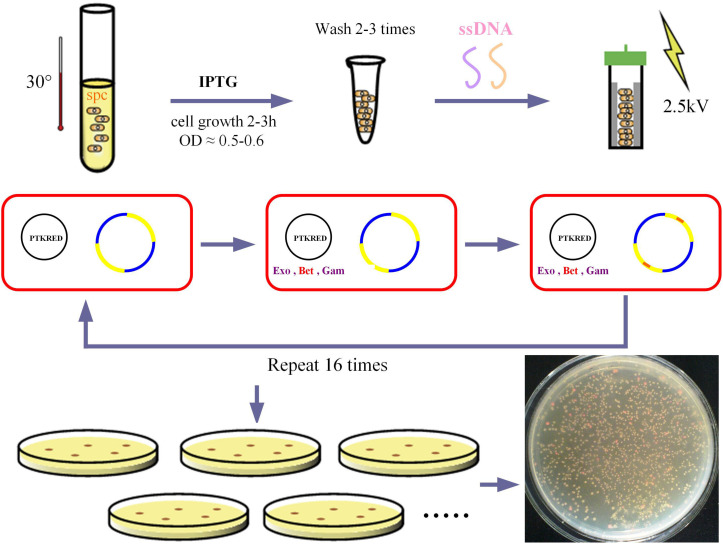
MAGE procedure. The red squares represent the cells, the black circles represent the ptkRED plasmid, and Exo, Bet and gam are the gene sequences of the enzymes on the plasmid. The yellow and blue circles represent the bacterial genome, with the red portion representing the target engineered gene sequence. Exo, exonuclease; Bet, phage recombination protein Bet; gam, host-nuclease inhibitor protein Gam.

After 16 rounds of MAGE modulation (Figure 3), recombinant strains were screened by Nile red staining. Recombinant strains with higher PHB production showed redder color. Screening and sequencing results are shown in Table 3. Using single colony color screening, we found that the RBS of both *glk* and *galP* genes were changed, and the amount of ssDNA (single string DNA) recombination of *glk* exceeded that of *galP*. This may be because the location of the *glk* gene is more susceptible to ssDNA recombination during genome replication. However, no recombinant was screened out in which the two genes were simultaneously mutated.

**TABLE 3:** Screening result of MAGE recombination strains.

Strain number	Gene	Origin RBS sequence	Mutated RBS sequence
1	*glk*	GGAGCAGTTGA	GAAGGGAGAGG
2	*glk*	GGAGCAGTTGA	GAAAGAAATGA
6	*galP*	TATTGGAGGGC	TGAAAGGGAAA
9	*glk*	GGAGCAGTTGA	GGAGGGATGGA
16	*glk*	GGAGCAGTTGA	TAGGAGGAGTT
18	*glk*	GGAGCAGTTGA	AAAAAGGGTTA
19	*glk*	GGAGCAGTTGA	GAAGGAGGGGT
20	*galP*	TATTGGAGGGC	GGAGAGGGTTA
21	*glk*	GGAGCAGTTGA	TGGGGGGAGGG
22	*glk*	GGAGCAGTTGA	AAAGGGGTTTG
23	*glk*	GGAGCAGTTGA	GAAGGGGTTTG
24	*glk*	GGAGCAGTTGA	AGAGGAAGAGA
27	*glk*	GGAGCAGTTGA	AAAAGGGATAG
28	*glk*	GGAGCAGTTGA	TTGGAAGATAT
30	*glk*	GGAGCAGTTGA	TGAGGAATGAA
32	*glk*	GGAGCAGTTGA	GTGGAAATAGA
36	*glk*	GGAGCAGTTGA	TTAGGGGGAGT
51	*glk*	GGAGCAGTTGA	TTAAGGGATAT
62	*glk*	GGAGCAGTTGA	GGAAGGAGAAT

**FIGURE 4:**
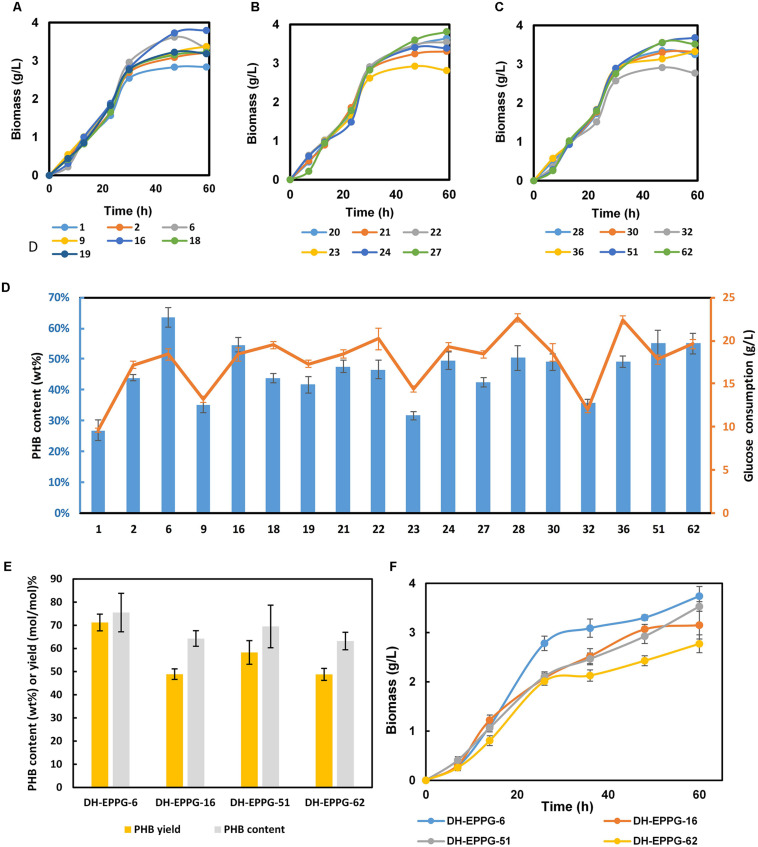
Fermentation result of the engineered EP-bifido strains. **(A)** Growth curve of first batch selection. **(B)** Growth curve of second batch selection. **(C)** Growth curve of third batch selection. **(D)** PHB content and glucose consumption of these three batches selection. The blue columns represent PHB content and orange red broken line represents glucose consumption. **(E)** PHB content and yield of selected recombinants. The experiments were performed in triplicate and error bars indicate s.d. For mutant fermentation selection, strains were cultivated with M9 at 37°C, 150 rpm. **(F)** Growth curve of selected recombinants.

Nineteen mutant strains were selected and transformed with pFF and pCAB, subsequently. Three batches fermentation of these 19 strains lead to the selection of strains in consideration of glucose consumption and PHB content, named EPPG-6, EPPG-16, EPPG-51, and EPPG-62 (Figure 4D). Then, we repeated fermentation using the four selected strains (Figure 4E). Fermentation results showed that after glucose transport system modulation, the DH-EPPG-6 strain had a recovered growth rate. The PHB yield reached 71.2% (mol/mol), which was 63.7% higher than that of the strain DH-EP. Then DH-EPPG-6 strain was renamed L-6, in which the wild type RBS sequence of *galP* was mutated to TGAAAGGGAAA.

### Expressing Heterogeneous Sugar Transporter to Reinforce Glucose Uptake

**FIGURE 5:**
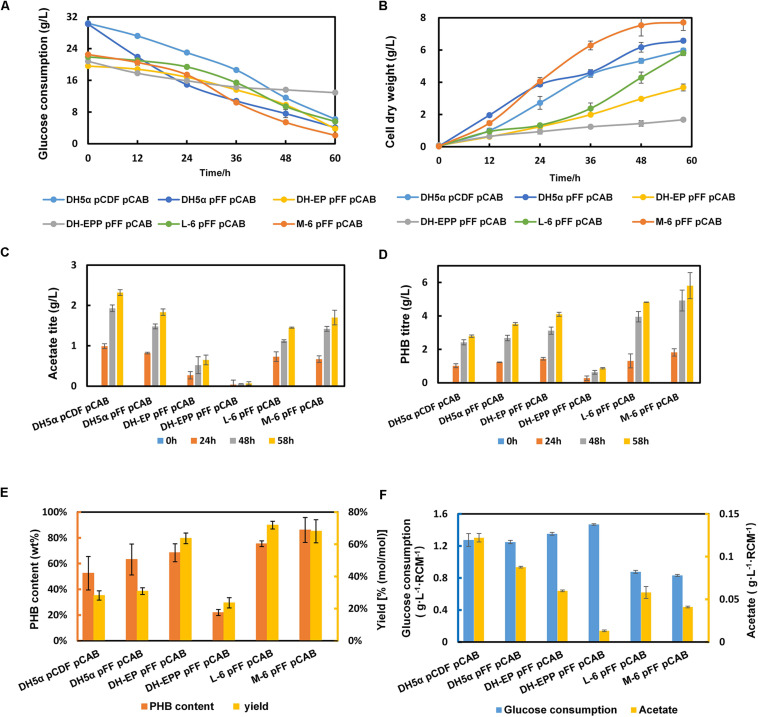
The fermentation profile and PHB yield of all the engineered EP-bifido strains. **(A)** Glucose consumption. **(B)** Cell dry weight. **(C)** Acetate production. **(D)** PHB production. **(E)** PHB content and PHB yield. **(F)** Glucose consumption and acetate normalized by residual cell mass. The experiments were performed in triplicate and error bars indicate s.d. Strains were cultivated in M9 with glucose at 37°C, 150 rpm.

**FIGURE 6:**
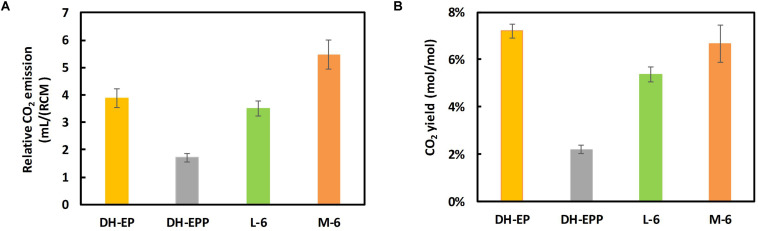
Determination of CO_2_ release. **(A)** Relative CO_2_ emission and **(B)** CO_2_ yield. Strains were cultivated in modified M9 medium containing 20 g/L glucose at 37°C and 150 rpm. CO_2_ yield were calculated as mol/mol glucose × 100%. The experiments were performed in triplicate and error bars indicate s.d.

To further enhance the consumption of glucose, we compared the kinetic parameters of several transporters with their glucose transport capacity and energy consumption during glucose internalization and phosphorylation (Gosset, 2005). Because the transmembrane proton potential is a form of energy, the energy consumption of the *glf* sugar transporter from *Zymomonas mobilis* is comparable to that of the *E. coli* glucose-specific PTS (PTS^Glc^). Compared with *galP*, the *glf*_*zm*_ transporter used less energy to produce a higher maximum velocity. Therefore, the high-rate, low-energy sugar transporter *glf*_*zm*_ was chosen to improve the glucose absorption capacity of engineered bacteria. Then *glf*_*zm*_ was inserted at the attP genomic site of strain L-6 using the CRIM plasmid system (Haldimann and Wanner, 2001), resulting the strain M-6. We deduced that *glf* integration was conducive to growth recovery in later growth stages. In Figure 5B, M-6 (pFFpCAB) showed a better growth curve than the control DH5α (pCDFpCAB). Simultaneously, as we expected, gross glucose consumption improved compared with L-6, increased from 16.2 to 20.4 g/L (Figure 5A). The glucose consumption of all engineered strains changed obviously after every modification step. PHB titer of M-6 improved significantly, from 4.82 to 5.81 g/L in comparison with that of L-6 (Figure 5D), and PHB content in M-6 strain reached 85.1 wt% (Figure 5E). The only drawback was that PHB yield of M-6 reached 68.1% (mol/mol) slightly decreased compared with L-6 (Figure 5E). In general, compared with parental strain DH-EP (pFFpCAB), the PHB titer and content improved 41.71 and 24.41% in M-6, respectively. And compared with the control DH5α (pCDFpCAB), the PHB content and yield of M-6 improved 61.9 and 141.7% in M-6, respectively (Figure 5). All the engineered strains produced some amount of acetate, M-6 and L-6 produced less acetate than DH-EP strain. We also calculated the acetate formation and glucose consumption normalized by residual cell mass (RCM).

In order to confirm that the glucose consumption rates in our engineered strains are indeed improved, we calculated the specific growth rate and glucose utilization rates of DH-EP (pFFpCAB), DH5α-EPP (pFFpCAB), L-6 (pFFpCAB), and M-6 (pFFpCAB). After *ptsG* gene was deleted, the specific growth rate of DH-EPP decreased from 0.20 to 0.09 h^–1^, and the glucose utilization rate decreased from 0.78 to 0.41 g L^–1^ h^–1^ (Table 4). After *glf*_*zm*_ integration based on L-6, the specific growth rate of M-6 (pFFpCAB) increased obviously from 0.28 to 0.50 h^–1^, improved 42.8% compared to that of DH5α (pFFpCAB). The growth consumption rate recovered to 0.71 g L^–1^ h^–1^, 73% higher than DH-EP (pFFpCAB). M-6 and L-6 showed lower normalized glucose consumption. Thus M-6 and L-6 had improved PHB biosynthesis and PHB productivity since they produced less by-product acetate and consumed less glucose per residual cell mass (Figure 5F and Table 5).

**TABLE 4:** Specific growth rates and glucose utilization rates of DH5α, DH-EP (pFFpCAB), DH-EPP (pFFpCAB), L-6 (pFFpCAB), and M-6 (pFFpCAB).

Strains	Specific growth rate (h^–1^)	Specific glucose utilization rate (g⋅L^–1^⋅h^–1^)
DH5α (pFFpCAB)	0.35 ± 0.011	0.78 ± 0.02
DH-EP (pFFpCAB)	0.20 ± 0.020	0.41 ± 0.03
DH-EPP (pFFpCAB)	0.09 ± 0.002	0.23 ± 0.01
L-6 (pFFpCAB)	0.28 ± 0.018	0.52 ± 0.01
M-6 (pFFpCAB)	0.50 ± 0.026	0.71 ± 0.03

*Specific growth rates and glucose utilization rates were calculated based on the 0–48 h data.*

**TABLE 5:** PHB productivity of the engineered PHB-producing strains.

Strains	PHB titer (g⋅L^–1^⋅h^–1^)
DH5α (pCDF pCAB)	0.048 ± 0.07
DH5α (pFF pCAB)	0.061 ± 0.08
DH-EP (pFF pCAB)	0.071 ± 0.012
DH-EPP (pFF pCAB)	0.015 ± 0.04
L-6 (pFF pCAB)	0.083 ± 0.015
M-6 (pFF pCAB)	0.100 ± 0.078

We further examined the CO_2_ release from the constructed EP-bifido strains. The total CO_2_ release of DH5α-EPP (pFFpCAB) decreased to 32.5% compared to DH-EP (pFFpCAB) (Figure 6A). And the CO_2_ emission of L-6 (pFFpCAB), M-6 (pFFpCAB) improved 177 and 332% compared to that of DH5α-EPP (pFFpCAB). We believe that the restoring growth contributed to the increased CO_2_ release. The CO_2_ yield of DH5α-EPP (pFFpCAB), L-6 (pFFpCAB), and M-6 (pFFpCAB) decreased to 16.9% (mol/mol), 74.4% (mol/mol) and 92.4% (mol/mol) compared to their controls, respectively (Figure 6B). The above data confirmed the recovery of growth after PTS system deficiency and the decreased CO_2_ emission from the L-6 (pFFpCAB) and M-6 (pFFpCAB) strains.

## Discussion

As environmental problems intensify, carbon saving or carbon sequestration pathways have become a new focus for bio-manufacturing. Previously, we successfully constructed an efficient carbon-saving pathway in *E. coli* called the EP-bifido pathway. This pathway has been applied to the production of several compounds that use acetyl-CoA as a precursor. As a degradable material, PHB has great application prospects. While we believe that there is potential for further optimization of PHB production by the EP-bifido pathway. In this study, we knocked out the *ptsG* gene, a key glucose transporter of the PTS system that employs PEP as phosphate donor. The glucose consumption rate and cell growth were significantly reduced in the *ptsG* mutant under aerobic fermentation conditions. The deficiency in the PTS system dramatically impairs glucose uptake and causes growth restriction. It is speculated that the reason for growth restriction is not insufficient glucose uptake, but the subsequent decrease in glucose phosphorylation efficiency due to limited glucokinase activity (Steinsiek and Bettenbrock, 2012).

To overcome this growth hindrance, we optimized the RBS sequences of *galP* and *glk* genes, encoding glucose permease and glucokinase, respectively. Subsequently, we introduced the *Z. mobilis* glucose transporter, *glf*_*zm*_, into the L-6 high-yield strain, and observed cell growth recovery. After optimization, PHB yield reached 71.9% (mol/mol) in L-6 strain. In the resulting strain M-6, the intracellular PHB content reached 85.1 wt%, and the titer reached 5.81 g/L. Previously, most studies have compensated for PTS knockout-induced inhibition of glucose uptake by overexpression of *glk*, and *galP* or by heterologous expression of *glf* (Snoep et al., 1994; Gosset, 2005; Lin et al., 2018). Instead, we applied MAGE technology to directly alter genomic *glk* and *galP* to optimize their expression (Gallagher et al., 2014). Meanwhile, the RBS library constructed using MAGE provided rich genotypes for subsequent screening of high-yield PHB strains. After *glf*_*zm*_ integration, recovered growth rate and glucose consumption was evident in strain M-6. Compared with parent strain DH-EP (pFFpCAB), the glucose consumption of M-6 increased 4.6 g/L, 29.1% higher than that of parent strain in Figure 5A. The PHB content and yield of strain M-6 improved compared with that of DH-EP(pFFpCAB). M-6 and L-6 showed lower normalized glucose consumption. Thus M-6 and L-6 had improved PHB production since they produced less by-product acetate and consumed less glucose per residual cell mass. We inferred that increased glucose uptake enhanced flux through EMP, which is supported by increased cell growth. The growth of DH-EP strain improved with decreased acetate formation in M-6 strain. The specific growth rate and CO_2_ release data of constructed strains further confirmed our inference. Our study provided an efficient way for improving glucose absorption and total carbon conversion rate in artificial carbon-saving pathways by replacing PTS with other glucose transporters. It also describes an efficient screening strategy for MAGE ssDNA recombineering technology. The efficient utilization of carbon sources has been one of the determinant for high productivity in microbial fermentation. In the future, the effective allocation of carbon resources and the construction of effective strategies for balancing cell growth and product biosynthesis will still be the direction of metabolic engineering.

## Data Availability Statement

All datasets generated for this study are included in the article/supplementary material.

## Author Contributions

QW and QQ designed the work. YLi, YLu, and JX performed the experiments. ZS and YLi analyzed the ^13^C-MFA data. QQ and QL encouraged this project. YLi and QW wrote the manuscript. All authors read and approved the final manuscript. All authors contributed to the article and approved the submitted version.

## Conflict of Interest

The authors declare that the research was conducted in the absence of any commercial or financial relationships that could be construed as a potential conflict of interest.

## References

[B1] Baba T. Ara T. Hasegawa M. Takai Y. Okumura Y. Baba M. (2006). Construction of *Escherichia coli* K-12 in-frame, single-gene knockout mutants: the Keio collection. *Mol. Syst. Biol.* 2:2006.0008. 10.1038/msb4100050 PMC1681482 16738554

[B2] Bogorad I. W. Lin T. S. Liao J. C. (2013). Synthetic non-oxidative glycolysis enables complete carbon conservation. *Nature* 502 693–697. 10.1038/nature12575 24077099

[B3] Datsenko K. A. Wanner B. L. (2000). One-step inactivation of chromosomal genes in *Escherichia coli* K-12 using PCR products. *Proc. Natl. Acad. Sci. U.S.A.* 97 6640–6645. 10.1073/pnas.120163297 10829079 PMC18686

[B4] Erb T. J. Berg I. A. Brecht V. Muller M. Fuchs G. Alber B. E. (2007). Synthesis of C5-dicarboxylic acids from C2-units involving crotonyl-CoA carboxylase/reductase: the ethylmalonyl-CoA pathway. *Proc. Natl. Acad. Sci. U.S.A.* 104 10631–10636. 10.1073/pnas.0702791104 17548827 PMC1965564

[B5] Gallagher R. R. Li Z. Lewis A. O. Isaacs F. J. (2014). Rapid editing and evolution of bacterial genomes using libraries of synthetic DNA. *Nat. Protoc.* 9 2301–2316. 10.1038/nprot.2014.082 25188632

[B6] Gong F. Cai Z. Li Y. (2016). Synthetic biology for CO2 fixation. *Sci. China Life Sci.* 59 1106–1114. 10.1007/s11427-016-0304-2 27787752

[B7] Gosset G. (2005). Improvement of *Escherichia coli* production strains by modification of the phosphoenolpyruvate:sugar phosphotransferase system. *Microb. Cell Fact* 4:14. 10.1186/1475-2859-4-14 PMC1156936 15904518

[B8] Haldimann A. Wanner B. L. (2001). Conditional-replication, integration, excision, and retrieval plasmid-host systems for gene structure-function studies of bacteria. *J. Bacteriol.* 183 6384–6393. 10.1128/jb.183.21.6384-6393.2001 11591683 PMC100134

[B9] Hernandez-Montalvo V. Martinez A. Hernandez-Chavez G. Bolivar F. Valle F. Gosset G. (2003). Expression of galP and glk in a *Escherichia coli* PTS mutant restores glucose transport and increases glycolytic flux to fermentation products. *Biotechnol. Bioeng.* 83 687–694. 10.1002/bit.10702 12889033

[B10] International Energy Agency (2019). *Data From: Global Energy and CO_2__Status Report.* Paris: International Energy Agency.

[B11] Kuhlman T. E. Cox E. C. (2010). Site-specific chromosomal integration of large synthetic constructs. *Nucleic Acids Res.* 38:e92. 10.1093/nar/gkp1193 20047970 PMC2847246

[B12] Kyselova L. Kreitmayer D. Kremling A. Bettenbrock K. (2018). Type and capacity of glucose transport influences succinate yield in two-stage cultivations. *Microb. Cell Factor.* 17:132. 10.1186/s12934-018-0980-1 PMC6112142 30153840

[B13] Lee J. W. Na D. Park J. M. Lee J. Choi S. Lee S. Y. (2012). Systems metabolic engineering of microorganisms for natural and non-natural chemicals. *Nat. Chem. Biol.* 8 536–546. 10.1038/nchembio.970 22596205

[B14] Lee S. J. Lee D. Y. Kim T. Y. Kim B. H. Lee J. W. Lee S. Y. (2005). Metabolic engineering of *Escherichia coli* for enhanced production of succinic acid, based on genome comparison and in silico gene knockout simulation. *Appl. Environ. Microbiol.* 71 7880–7887. 10.1128/aem.71.12.7880-7887.2005 16332763 PMC1317394

[B15] Lee S. Y. Choi J. I. (2001). Production of microbial polyester by fermentation of recombinant microorganisms. *Adv. Biochem. Eng. Biotechnol.* 71 183–207. 10.1007/3-540-40021-4_6 11217412

[B16] Lee S. Y. Lee K. M. Chang H. N. Steinbuchel A. (1994). Comparison of recombinant *Escherichia-Coli* strains for synthesis and accumulation of poly-(3-hydroxybutyric acid) and morphological-changes. *Biotechnol. Bioeng.* 44 1337–1347. 10.1002/bit.260441110 18618646

[B17] Li Y. Li M. Zhang X. Yang P. Liang Q. Qi Q. (2013). A novel whole-phase succinate fermentation strategy with high volumetric productivity in engineered *Escherichia coli*. *Bioresour. Technol.* 149 333–340. 10.1016/j.biortech.2013.09.077 24125798

[B18] Li Z. J. Cai L. Wu Q. Chen G. Q. (2009). Overexpression of NAD kinase in recombinant *Escherichia coli* harboring the phbCAB operon improves poly(3-hydroxybutyrate) production. *Appl. Microbiol. Biotechnol.* 83 939–947. 10.1007/s00253-009-1943-6 19357844

[B19] Lin P. P. Jaeger A. J. Wu T. Y. Xu S. C. Lee A. S. Gao F. K. (2018). Construction and evolution of an *Escherichia coli* strain relying on nonoxidative glycolysis for sugar catabolism. *Proc. Natl. Acad. Sci. U.S.A.* 115 3538–3546. 10.1073/pnas.1802191115 29555759 PMC5889684

[B20] Meile L. Rohr L. M. Geissmann T. A. Herensperger M. Teuber M. (2001). Characterization of the D-xylulose 5-phosphate/D-fructose 6-phosphate phosphoketolase gene (xfp) from *Bifidobacterium lactis*. *J. Bacteriol.* 183 2929–2936. 10.1128/jb.183.9.2929-2936.2001 11292814 PMC99511

[B21] Mozumder M. S. I. De Wever H. Volcke E. I. P. Garcia-Gonzalez L. (2014). A robust fed-batch feeding strategy independent of the carbon source for optimal polyhydroxybutyrate production. *Process Biochem.* 49 365–373. 10.1016/j.procbio.2013.12.004

[B22] Posthuma C. C. Bader R. Engelmann R. Postma P. W. Hengstenberg W. Pouwels P. H. (2002). Expression of the xylulose 5-phosphate phosphoketolase gene, xpkA, from Lactobacillus pentosus MD363 is induced by sugars that are fermented via the phosphoketolase pathway and is repressed by glucose mediated by CcpA and the mannose phosphoenolpyruvate phosphotransferase system. *Appl. Environ. Microbiol.* 68 831–837. 10.1128/aem.68.2.831-837.2002 11823225 PMC126734

[B23] Ragauskas A. J. Williams C. K. Davison B. H. Britovsek G. Cairney J. Eckert C. A. (2006). The path forward for biofuels and biomaterials. *Science* 311 484–489. 10.1126/science.1114736 16439654

[B24] Schwander T. Schada Von Borzyskowski L. Burgener S. Cortina N. S. Erb T. J. (2016). A synthetic pathway for the fixation of carbon dioxide in vitro. *Science* 354 900–904. 10.1126/science.aah5237 27856910 PMC5892708

[B25] Snoep J. L. Arfman N. Yomano L. P. Fliege R. K. Conway T. Ingram L. O. (1994). Reconstruction of glucose uptake and phosphorylation in a glucose-negative mutant of *Escherichia coli* by using Zymomonas mobilis genes encoding the glucose facilitator protein and glucokinase. *J. Bacteriol.* 176 2133–2135. 10.1128/jb.176.7.2133-2135.1994 8144485 PMC205325

[B26] Steinsiek S. Bettenbrock K. (2012). Glucose transport in *Escherichia coli* mutant strains with defects in sugar transport systems. *J. Bacteriol.* 194 5897–5908. 10.1128/jb.01502-12 22923596 PMC3486086

[B27] Valle F. Munoz E. Ponce E. Flores N. Bolivar F. (1996). Basic and applied aspects of metabolic diversity: the phosphoenolpyruvate node. *J. Industr. Microbiol. Biotechnol.* 17 458–462. 10.1007/bf01574776

[B28] van der Walle G. A. De Koning G. J. Weusthuis R. A. Eggink G. (2001). Properties, modifications and applications of biopolyesters. *Adv. Biochem. Eng. Biotechnol.* 71 263–291. 10.1007/3-540-40021-4_9 11217415

[B29] Wang H. H. Isaacs F. J. Carr P. A. Sun Z. Z. Xu G. Forest C. R. (2009). Programming cells by multiplex genome engineering and accelerated evolution. *Nature* 460 894–898. 10.1038/nature08187 19633652 PMC4590770

[B30] Wang Q. Yu H. M. Xia Y. Z. Kang Z. Qi Q. S. (2009). Complete PHB mobilization in *Escherichia coli* enhances the stress tolerance: a potential biotechnological application. *Microb. Cell Factor.* 8:47. 10.1186/1475-2859-8-47 19719845 PMC2746179

[B31] Wang Q. Wu C. Chen T. Chen X. Zhao X. (2006). Expression of galactose permease and pyruvate carboxylase in *Escherichia coli* ptsG mutant increases the growth rate and succinate yield under anaerobic conditions. *Biotechnol. Lett.* 28 89–93. 10.1007/s10529-005-4952-2 16369691

[B32] Wang Q. Xia Y. Z. Chen Q. Qi Q. S. (2012). Incremental truncation of PHA synthases results in altered product specificity. *Enzyme Microb. Technol.* 50 293–297. 10.1016/j.enzmictec.2012.02.003 22500895

[B33] Wang Q. Xu J. Sun Z. Luan Y. Li Y. Wang J. (2019). Engineering an in vivo EP-bifido pathway in *Escherichia coli* for high-yield acetyl-CoA generation with low CO_2_ emission. *Metab. Eng.* 51 79–87. 10.1016/j.ymben.2018.08.003 30102971

